# Periscope

**Published:** 1889-06

**Authors:** 


### PERISCOPE.

### MEDICINE.

**The Juvenile Form of Progressive Muscular Atrophy.**—First recognised by Erb in 1884 as a clinically distinct type, this form includes those cases beginning usually at puberty, and affecting the muscles of the shoulder chiefly—the "scapulo-humeral type." To our knowledge of this rare disease Professor Hitzig, of Halle, contributes four cases.

*Case I.* was a land-steward, 24 years of age. He complained of increasing weakness for nine months, and for six months had fibrillary contractions in the muscles of the upper limbs. No hereditary influence could be determined, nor could any cause be assigned for the onset. On inspection there was no atrophy; but on the contrary a great number of the muscles connected with the shoulder-girdle presented a markedly hypertrophic appearance, especially on the right side. In the middle part of the deltoid and in the triceps on the left side fibrillary tremors were observed. The motor power was less in the upper arm. The electric examination showed a distinct increase of intra- and extra-muscular irritability. An excised piece of affected muscle showed microscopically the fibres to be much hypertrophied and the sarcolemma nuclei increased.

*Case II.*—A man-servant, 19 years old. No hereditary history; complained of pain and impaired motion in the right elbow for six months. He showed a rigid contraction of the right biceps: on both sides pectoralis major was markedly developed, on the right side the clavicular portion of the muscle being strongly atrophic. There was an alteration in the relation of the scapula to the chest-wall; calves were normal. There was a slight increase in the intra-muscular irritability in the right biceps; but it was entirely lost in the middle and upper portions of the pectorals. The Galvanic current gives similar results. Microscopically the fibres showed moderate hypertrophy—no atrophied fibres being present.

*Case III.* was a girl, 21 years of age, well built and well nourished. The disease was said to arise from a blow seven years previous. From that time the right arm gradually became weaker—the left becoming affected two years later. She complained of tingling in the fingers of the right hand. She presented a characteristic picture of Erb's "juvenile dystrophy": pronounced hypertrophy was nowhere to be seen, although both deltoids were strongly developed and the calves voluminous. The usually affected muscles of the shoulder-girdle were strongly atrophic, with corresponding decrease of the Faradic excitability. Fibrillary tremors were observed on the patient's admission, but were transient. Pieces of muscle were examined from different parts of the greater pectoral. Where the muscle appeared atrophied, the fibres were of moderate size—some hypertrophic, others atrophic,—and between the fibres an amount of fat and connective tissue. In the apparently healthy part the majority of the fibres showed hypertrophy more or less.

*Case IV.* was a man, *ætat.* 46. No heredity; drank freely; affection began fifteen months before; complained of weakness in the arms and formication. He was a strongly-built man; The tongue deviated slightly to the right. There was a high degree of atrophy of the shoulder and upper arm region. Microscopically the fibres showed hypertrophy; some, longitudinal atrophy; while in other fibres circular atrophy obtained.

These cases support the proposition of Barsickow, that the hypertrophy of the fibres represents an early stage of the atrophic condition. Case IV. resembled the spinal form in that paræsthesiæ occurred early; it ran a more rapid course; the muscles of the hand were affected, and motor symptoms—*e.g.* cramps, increased reflex activity—were present. But the anatomical condition—the enormous hypertrophy of the fibres in conjunction with atrophic states—indicated the muscular form. In the spinal form there is a simple degenerative atrophy. It has also been shown that the early affection of the muscles of the hand does not possess the importance which Erb attached to it. Fibrillary tremors, as in I. and III., are not usually present, while paræsthesiæ and increased reflexes do sometimes occur in genuine cases of "juvenile dystrophy." Hitzig points out that the wing-like position of the scapula supposed to be characteristic of serratus paralysis may occur in this disease—*e.g.* Case III. (where the serratus still reacted)—as indeed it may take place in affection of the rhomboidei and the lower and middle portions of the trapezius. The essential nature of the juvenile atrophy consists, not in any interstitial process, but in a parenchymatous change which shows itself chiefly in a lesser or greater degree of hypertrophy according to the intensity of the disease, thus differing fundamentally from pseudo-hypertrophic paralysis, where the change is one affecting primarily the connective tissue; and with regard to the differential diagnosis between the muscular and spinal forms of progressive muscular atrophy Hitzig asserts that in many cases it is only possible through a microscopical examination.—*Berliner klinische Wochenschrift*, Nos. 25, 34 and 35, 1888.

**Remarkable Case of Cancer of the Dura Mater.**—Loewenmeyer recently brought before the Berlin Medical Society a remarkable pathological preparation. The patient from whom the specimen was obtained was a man of 44 years of age. He had sickened some months before with cough, expectoration, and pain in the side. This increased very much, and he was admitted into the Jewish Hospital. There was a serous exudation in the right pleura and infiltration of the right lung. There were no tubercle-bacilli. Malignant disease was suspected. At the autopsy cancer of the right lung was found, a number of nodules being scattered throughout the lung-substance, which was also infiltrated. The left lung was healthy. Besides this there were discovered two cancerous tumours of the dura mater, both about the size of a walnut, lying symmetrically on either side of the longitudinal sinus with their base resting on the dura mater. They had grown through the bones of the skull, forming two circular holes; one, in fact, having grown into the scalp and produced a small elevation there. There was absolutely no sign of brain-affection during life. Professor Virchow remarked on the great interest of the case, and mentioned that both the tumours and the secondary growths in the lungs were epithelial. He attributed the absence of brain-symptoms to the outward growth of the tumours.—*Berliner klinische Wochenschrift*, No. 44, 1888.

**A Case of Cocaine Poisoning.**—Haenol details a case of a girl, nineteen years of age, in whom a dentist injected into the gum three-quarters of a syringeful of 15 per cent. cocaine solution—that is, about 1½ grains in two portions one after the other. The tooth was extracted, but soon after the patient became pale and fell down, being seized with violent convulsions broken by short pauses. When Haenol saw her she was unconscious, groaning, and could not be roused. The trunk and extremities were affected by clonic spasms, which lasted five hours. Face was not affected; there was no exophthalmos; pupils were moderately wide and reactionless. Temperature at the end of this stage 100.8° F. Pulse could not be counted at first, but later numbered 176 beats.

Respiration was 44. After spasms ceased, the patient lay unconscious for two hours. When she came to, she was unable to stand, could not lift her arm nor press the hand; she had intense photophobia, lessened sensation in the skin, anæsthesia of mucous membrane of nose and mouth, loss of smell and taste, dryness and burning in the throat, thirst and great strangling. Pulse was now 137. Respiration 28. Then cardialgia set in, and lasted for six days, being very severe. Retention of urine for twenty-four hours, sleeplessness for thirty hours, and complete loss of appetite for four days were also observed. Nitrite of amyl, large doses of opium, cold application to head were tried with no success. There was no previous history of fits. The above symptoms coincide with what we know of the pharmacological action of cocaine. Thus Feinberg observed in animals complete anæsthesia, unconsciousness, epileptiform spasms of cortical origin caused by vaso-motor spasm and anæmia of the cortex. Von Anrep found a great increase in the blood-pressure and marked quickening of the pulse, due to stimulation of the accelerative fibres of the heart—not paralysis of the vagus—and also diminution of the mucous secretions. — *Berliner klinische Wochenschrift*, No 44, 1888. D. R. PATERSON, M.D.

**A Clinical Study of Acromegalie.**—Dr. P. Marie, availing himself of the accounts of additional cases of this disease that have recently been published, and of the results of an autopsy on one of his patients, gives a more complete account of the changes found than he was able to do in his first memoir on the subject.

The most striking characteristic of the disease is the hypertrophy of the extremities, the head, the hands and feet. The hands are enormous, their general form regular but flat, and their size out of proportion to their length. The fingers sausage-shaped, flattened antero-posteriorly; the articulation between the 2nd and 3rd terminal phalanges swollen. The folds of the hands are exaggerated and bordered by enormous pads. Towards the inner part of the hypothenar eminence is a large mass of flesh which can be readily separated from the 5th metacarpal bone. The nails are flattened, short, and broad, and show longitudinal striation. The soft parts, as well as the bony skeleton, take part in the enlargement of the hands. The thumb is generally a little increased in size, but not to the same extent as the hand. The arm and fore arm remain unaffected.

The feet present corresponding changes: they are flat and very large, and on the outer border a thick mass of flesh forms an enormous pad. The malleoli are more or less enlarged, and the patella and condyles of the femur are also increased in size: the thigh and leg are natural.

The vault of the skull preserves its normal shape and size little changed. The face is very much elongated. The forehead low, with overhanging brows from dilatation of the frontal sinuses. The eyelids are long, sometimes thickened, and with hypertrophy of the tarsal cartilages. The nose is enormous, increased in all its dimensions. The cheeks flat and long, with prominent cheek-bones. The lower lip is very large, prominent and protruding: this increase in size of the lower lip contributes largely to the characteristic aspect of the patient. The chin is prominent, large and massive; the lower maxilla much increased in size; and, as the upper lip does not share in this increase, the face is prognathous often to a marked degree. The general shape of the face is an elongated oval. The teeth undergo no change, except that from the growth of the lower jaw they are here a little separated one from the other. The tongue attains very large dimensions, but keeps its normal shape. The ears are sometimes, but not always, increased in size.

As for the spine, if the affection is pronounced, there is very marked kyphosis in the upper dorsal, with compensating lordosis in the lumbar region. There may be a certain degree of scoliosis, and the vertebræ themselves are found to be hypertrophied. The neck is large, but short. From the shortness of the neck, the length of the chin, and kyphosis, the head is sunk between the shoulders and carried forwards, so that the chin may almost rest upon the sternum.

In the chest the clavicles, sternum, and ribs may be enlarged; the circumference of the thorax is great; the ribs oblique, their cartilages large. When the disease is well marked, the antero-posterior diameter of the thorax is increased, and its sides flattened. The xiphoid is enormous, projecting, with obliquity of the sternum from above downwards. In deep respiration the lower part of the thorax moves forward in a remarkable degree. Respiration is chiefly diaphragmatic.

The articulations are prominent, sometimes nodular. In an advanced stage of the disease the muscles are weak and small; but in the early stages muscular power is good, or even excessive. Electro-excitability of the muscles is said to be augmented. Symptoms are, briefly, headache, articular pains, sight feeble, or even blindness, and weakness. The skin is flabby, sometimes dry, and often presents a yellowish-brown discolouration, most marked on the eyelids.

The larynx is generally increased in size, and the voice strong and deep. The heart is believed to be frequently enlarged.

The mental state is normal. Sexual desire and power is generally lost, and in the female the catamenia cease.

The two sexes seem to be equally liable to the disease; the duration may be twenty or thirty years, or longer; the morbid changes slowly advance, with increasing weakness and a general cachectic state. The age of onset is at present uncertain.—*Le Progrès médical*, March 16th, 1889.

J. Michell Clarke, M.B.

### THERAPEUTICS.

**Local Treatment of Acne.**—Isaac considers the chief indication is, to limit the abundant vascularisation by the removal of the moisture and so cause shrinking. For this purpose Lassar's paste is chiefly used, consisting of—

Naphthol, 10 parts.
Sulph. Præcip., 50 parts.
Sapon. Viridis.
Vaselini āā, 20 parts.

This is spread upon the affected skin with a brush or spatula, and, according to the intensity of the process, allowed to remain on half-an-hour to one hour. On the following day scaling and retraction of the affected part, combined with slight irritation, may be seen. This is done every day until complete scaling of the skin has occurred. If severe irritation ensues, the treatment is stopped, and the part powdered, or a mild ointment, such as 2 per cent. salicyl. paste, used. For very obstinate cases the following is used:

Pulv. Cretæ Albæ, 5 parts.

β. Naphthol.

Camphor.

Vaselini flav., āā 10 parts.

Saponis Viridis, 15 parts.

Sulphur. Præcipit., 50 parts.

The addition of camphor increases the scaling action of the paste, which ought to be applied, however, for only a quarter of an hour to avoid irritation.

Another remedy of great use here is Resorcin:

Resorcin.
Zinci Oxydat.
Amyl, āā 5 parts.
Vaselini flav., 10 parts.
Ft. Pasta Mollis.

This produces a mild scaling of the skin. It was used in about 50 cases in 10—20 per cent. form without any disturbance. It is best, however, to begin with weaker applications, and gradually increase it to 20 per cent., in order to avoid untoward scaling. This treatment ought to be supplemented by the use of soaps, which must be used long after all appearance of acne has disappeared.—*Berliner klinische Wochenschrift*, No. 3, 1889.

**Hydracetin.**—This substance is the most active constituent of the drug called Pyrodin, which was investigated last year by Dreschfeld, and found to be a powerful antipyretic. The latter was shown to be an impure compound, and there has been separated from it an active principle which is four times as strong and to which the above name has been given. It is a white, crystalline, colourless, and almost tasteless powder, with difficulty soluble in water (1 in 50), but readily so in alcohol. It has recently been investigated by Dr. Paul Guttmann, who has embodied his results in a paper read before the Berlin Medical Society. In small doses (1½ to 2¼ grs.), hydracetin reduces fever-temperatures. It was tried in 18 cases—8 being enteric fever, 3 phthisis, 2 pneumonia, 2 scarlet fever, and one each of erysipelas, acute tuberculosis, and septicæmia. In the enteric cases it was given on different days; in most of the others only once. The doses varied from ¾ gr. to 3 grs.; the gross daily dose in the majority being 2¾ grs., divided into two or three doses. Usually 1½ grs. were first given, followed in two hours by one of ¾ gr. Half an hour after the first dose the temperature begins to fall, and continues doing so for two hours, or at most three, when it reaches its lowest level. The fall is usually 2½° to 3½° F.; in exceptional cases 5°. It is usually evanescent, the thermometer soon ascending and reaching its point prior to the administration about three or four hours after. By giving ¾ gr. every hour for three hours, it can be kept down for as long a period as six hours. The decrease of temperature is accompanied by more or less profuse sweating, while the pulse and respiration fall.

In acute rheumatism, hydracetin was tried in eight cases, being administered in doses of 1½ to 3 or 4½ grs. per day. Its action in relieving pain was unmistakable; it ensued mostly after the first dose, always after the second, and usually within half to one hour of the administration of the drug. The movements in the joints became greater; after a time the pains returned, to be directly relieved again by another powder. It is thus to be looked upon as a palliative remedy in acute joint-rheumatism, similar to the salicylates, antipyrin, antifebrin, &c. Its exhibition in two cases of sciatica was followed by immediate, though temporary, relief of the pain.

The powerful reducing action which hydracetin has chemically, suggested its use in psoriasis, where reducing agents, such as chrysarobin and pyrogallic acid, act so well. The inunction of a ten per cent. ointment removed a patch on the back after seven days, and in another instance it was used on the arm with perfect result.

Guttmann, while considering the drug worthy of a trial, does not recommend its more constant use in one case, as toxic symptoms may readily set in. Thus in three cases where $1\frac{1}{2}$ grs. were given twice daily for seven days, pallor of the face came on: one of the physiological results of large doses in lower animals is, destruction of the red cells of the blood.

The following rules are given for its administration: 1. The daily dose should not be higher than $1\frac{1}{2}$ grs. for adults; and, if used as an antipyretic, should be given at once, or in two doses with the interval of an hour. In acute rheumatism, $1\frac{1}{2}$ grs. divided into two may be prescribed—one half given in the forenoon, the other half in the afternoon. 2. Doses of $1\frac{1}{2}$ grs. daily should not be continued longer than three consecutive days. An interruption of several days should follow. —*Berliner klinische Wochenschrift*, No. 20, 1889.

D. R. Paterson, M.D.

**Treatment of Syphilis by Hypodermic Injections.** —Dr. Gelineau thinks that this is the most simple and effective plan of treating syphilis, and that it is free from the disadvantages that attend the administration of mercury by the mouth. He prefers a solution of the cyanide of mercury, as being always perfectly well tolerated. He gives two injections a week during the period of the appearance of the chancre and of the secondary manifestations of the disease on the skin and mouth. Fifteen days after their disappearance he begins injections of a solution of iodine in oil (1—20); these are given once a week for three months. Then 8—10 of the mercurial injections are given in a period of twenty days; the iodine injections are then continued for another period of three months, and in this way the two medicaments are given alternately during one year.—*La Médecine hypodermique*, April 1st, 1889.

J. Michell Clarke, M.B.

